# Prognostic Value of Liver Function and Tumor Burden Scores in Patients with HCC After Transarterial Radioembolization – Retrospective Single Center Analysis

**DOI:** 10.1007/s12029-026-01572-1

**Published:** 2026-08-13

**Authors:** Aaron Schindler, Anne Beeskow, Janett Fischer, Sebastian Ebel, Timm Denecke, Daniel Seehofer, Thomas Lincke, Osama Sabri, Thomas Berg, Florian van Bömmel

**Affiliations:** 1 Division of Hepatology, Department of Medicine II, Leipzig University Medical Center, Liebigstr. 20, 04103 Leipzig, Germany; 2 Department of Diagnostic and Interventional Radiology, Leipzig University Medical Center, Leipzig, Germany; 3 Department of Visceral, Vascular, Thoracic and Transplant Surgery, Leipzig University Medical Center, Leipzig, Germany; 4 Department of Nuclear Medicine, Leipzig University Medical Center, Leipzig, Germany; 5 Leipzig University Medical Center, University Cancer Center Leipzig (UCCL), Leipzig, Germany

**Keywords:** Liver cancer, Locoregional therapy, Liver function

## Abstract

**Background:**

Transarterial radioembolization (TARE) is an established locoregional therapy for advanced hepatocellular carcinoma (HCC). Prognosis depends on both tumor progression and hepatic function. Despite multiple scoring systems, predicting therapeutic benefit remains challenging.

**Materials and Methods:**

This study evaluated the prognostic performance of established scores (BCLC, HAP, CLIP, OKUDA, ALBI, MELD, and Child-Pugh) for survival after TARE in 86 patients with HCC and liver cirrhosis. Predictive accuracy for 3-, 6-, and 12-month survival was assessed using ROC curve analyses.

**Results:**

The median age was 67 years, and the predominant etiologies were alcohol-related and metabolic dysfunction–associated steatotic liver disease. Only ALBI and OKUDA were significantly associated with overall survival (HR 3.10; *p* < 0.001 and HR 5.02; *p* = 0.018, respectively). ALBI and CLIP showed the highest predictive accuracy for 3-, 6-, and 12-month survival. In multivariablee analysis, ALBI and OKUDA remained independent predictors of 12-month survival (HR 3.70; *p* = 0.001 and HR 5.01; *p* = 0.025, respectively). The ALBI score worsened significantly within 12 weeks after TARE (*p* < 0.001).

**Conclusion:**

The ALBI score was the strongest predictor of survival following TARE. In contrast, tumor burden showed limited prognostic relevance, underscoring hepatic functional reserve as a key determinant of outcomes after locoregional liver cancer therapy.

**Supplementary Information:**

The online version contains supplementary material available at 10.1007/s12029-026-01572-1.

## Introduction

Hepatocellular carcinoma (HCC) is the most common form of primary liver cancer and represents the third leading cause of cancer-related mortality worldwide [1, 2]. Treatment strategies are determined by disease stage and include surgical resection, locoregional therapies and systemic treatments [3–5]. While transarterial chemoembolization (TACE) remains the standard of care for most patients with intermediate-stage HCC, transarterial radioembolization (TARE) has emerged as an effective alternative in selected cases. Current Barcelona Clinic Liver Cancer (BCLC) guidelines recommend TARE for patients in very early (0), early (A), or intermediate (B) HCC stages [6], whereas its use in stage C is not supported due to insufficient evidence from randomized trials [3, 4].

Although some uncertainty remains regarding the exact position of TARE within the therapeutic algorithm of HCC, recent studies have demonstrated high efficacy and favorable safety in well-selected patients, with several analyses suggesting superior outcomes compared with TACE [7, 8]. Compared with other locoregional therapies, TARE can be safely applied in patients with portal vein invasion, making it a valuable treatment option in this clinical setting.

Despite limited evidence from prospective phase III trials, TARE has demonstrated effectiveness in numerous patients with unresectable HCC. Although it has not been proven superior to sorafenib in randomized trials, a growing body of research suggests TARE is non-inferior to sorafenib, with better tolerability [9–12].

Although randomized studies have not consistently demonstrated an overall survival benefit of TARE over standard treatment, and radiation-induced liver toxicity remains a relevant concern, TARE offers several important advantages. These include high objective response rates, feasibility in carefully selected patients with portal vein tumor thrombosis, and the potential to achieve downstaging to resection or liver transplantation in a substantial proportion of patients. Therefore, current guidelines recommend careful patient selection and multidisciplinary decision-making when considering TARE [13–15].

Accurate prognostication is essential for appropriate treatment allocation in HCC. Several scoring systems assess liver function, tumor burden, or both. Commonly used liver function scores include the Child-Pugh-Turcotte (CPT) score, the Model for End-Stage Liver Disease (MELD) score and the albumin-bilirubin (ALBI) score [16–19]. The ALBI score, introduced in 2014, offers an objective, evidence-based method for assessing liver function and has been shown to provide superior prognostic value over CPT in HCC patients, particularly in those undergoing TARE [20–22]. In addition to liver function, several scores combine both liver function and tumor parameters to predict prognosis, such as the Okuda score, the Cancer of the Liver Italian Program (CLIP) score, and the hepatoma arterial-embolization prognostic (HAP) score [23–25].

The Okuda score, developed to assess prognosis in HCC, incorporates tumor size, ascites, and serum albumin and bilirubin levels, categorizing patients into three stages with corresponding survival estimates [25]. The CLIP score, which is specifically designed for patients with cirrhosis and HCC, includes Child-Pugh stage, tumor morphology, serum alpha-fetoprotein (AFP) levels, and portal vein thrombosis [23]. The HAP score, introduced in 2013, integrates serum albumin, bilirubin, AFP levels, and tumor size to predict survival in HCC patients undergoing TACE [24].

Given the variety of available prognostic tools and the lack of consensus on which score best predicts outcomes for patients undergoing TARE, further comparative evaluations are essential. Identifying reliable predictors of survival is of paramount clinical importance to optimize treatment strategies for HCC patients.

This study therefore aimed to evaluate the prognostic performance of these commonly used scoring systems in a real-word cohort of patients with HCC and cirrhosis treated with TARE.

## Patient and Methods

### Patient Selection

Between 2010 and 2020, 179 patients with HCC were treated with TARE with Y-90 microspheres at Leipzig University Medical Center based on a multidisciplinary tumor board decision. From 179 patients treated with TARE data were available for 86 patients (Fig. S1), cases were excluded due to a lack of availability of imaging (our tertiary center started storing radiological images automatically not until 2020). Inclusion criteria were a definite diagnosis of HCC by either histological or radiological criteria available follow-up data, patient age > 18 years, and availability of score-related parameters tumor size and burden, macrovascular invasion, portal vein thrombosis, ascites, AFP level, bilirubin and albumin levels. These parameters were determined to apply the prognostic scoring systems MELD, CPT, BCLC, ALBI, Okuda, CLIP, and HAP (Table S1).

Written informed consent was obtained from all patients. This study was approved by the Ethics Committees of Medical Research of the University of Leipzig in accordance with the Declaration of Helsinki of 1975 (revision 2013) and the International Conference on Harmonization/Committee for Proprietary Medicinal Products “Good Clinical Practice” guidelines (Vote No. 132/23-ek).

Response was assessed by imaging at 10–12 weeks after TARE by modified Response Evaluation Criteria in Solid Tumors (mRECIST) criteria based on either contrast-enhanced multiphasic computed tomography (CT) or magnetic resonance imaging (MRI).

### TARE Treatment

TARE was performed under local anesthesia. Probing was performed with a 4–5 F macrocatheter and a 2.0–2.7 F microcatheter. Each TARE was proceeded by diagnostic angiography with protective embolization of branches, if necessary, and finally lobar application of Tc-99 micro-aggregated albumin. Patients with a pulmonary shunt greater than 20% on positron emission tomography (PET) scan were excluded. For TARE, Y-90 microspheres were applied in the same position as for evaluation.

Patients received either glass-based (TheraSpheres^®^ Boston Scientific, Marlborough, Massachusetts) (*n* = 74) or resin-based (Sir-Spheres^®^ Sirtex Medical Limited, Sydney, Australia) (*n* = 12) microspheres. The mean administered dose of Y-90 was 2.86 ± 1.48 (range, 0.54-7.0) gigabecquerel (GBq).

### Statistical Analysis

Statistical analyses were performed using SPSS 27 (IBM Research, Armonk, USA) and R version 4.2.3. Values are presented as median and interquartile range unless otherwise specified. Categorical variables are reported as frequencies and percentages. Overall survival was plotted with a Kaplan-Meier curve. Friedman test and pairwise Wilcoxon signed-rank test were used to compare variables before TARE and after TARE. The performance of different scores to estimate 3-, 6- and 12-month survival was analyzed using time-dependent receiver operating characteristic (ROC) analysis. Uni- and multivariable Cox regression analysis of the following parameters were performed: age, sex, portal vein thrombosis, baseline levels of AFP, macrovascular infiltration, tumor burden, ALBI score, CLIP score, HAP score, Okuda score, CPT score, BCLC score and MELD score to detect associations between the variables and 3-, 6-, 12-month and overall survival. Multivariable Cox regression analysis was performed by using *p* < 0.05 for inclusion and *p* > 0.1 for exclusion of parameters in the final model. Variance inflation factor (VIF) was calculated to measure multicollinearity between the variables. Variables that produced VIF values greater than 2.5 were removed from the model. Hazard ratios (HR) and 95% confidence intervals (CI) were calculated. A p-value < 0.05 was considered statistically significant.

## Results

Of the 86 included patients 73 were male (84.9%) with a median age of 67 (range, 37–89) years. The etiology of HCC/liver cirrhosis was mainly alcoholic liver disease (ALD) or metabolic dysfunction-associated steatotic liver disease (MASLD) (51.2% and 14%, respectively). Other causes included chronic hepatitis C (4.7%), chronic hepatitis B (2.3%) and hemochromatosis (2.3%). BCLC stages were A, B or C in 2, 48, and 36 patients, respectively. CTP class was A in 93% and B in 7% of cases, respectively. Median ALBI score was − 2.74 (-3.47- -1.29), (patients’ characteristics and prognostic score results are listed in Table 1).

**Table 1:** Patient characteristics at the time of transarterial radioembolization (TARE)

Parameter	Patients (*n* = 86)
Male n (%)	73 (84.9%)
Age (years)*	67 (37–89)
Liver cirrhosis etiology n (%) ASH MASLD Cryptogenic HCV HBV Hemochomatosis AIH PBC Unknown	44 (51.2%) 13 (14%) 7 (8.1%) 4 (4.7%) 2 (2.3%) 2 (2.3%) 1 (1.2%) 1 (1.2%) 12 (12.9%)
Portal vein thrombosis (PVT) n (%)	34 (39.5%)
Macrovascular infiltration (MVI) n (%) PVT and MVI n (%)	28 (32.6%) 6 (7%)
Tumor size (cm)	7.5 (1.0-17.4)
Tumor burden (%) Tumor burden > 50% n (%)	20 (5–70) 17 (19.8%)
Multifocality	64 (74.4%)
Distant metastases	11 (12.8%)
BCLC stage n (%) A B C	2 (2%) 48 (56%) 36 (42%)
CTP class n (%) A B	80 (93%) 6 (7%)
ALBI score ALBI grade 1 n(%) ALBI grade 2 n (%) ALBI grade 3 n (%)	-2.74 (-3.47- -1.29) 50 (58.1%) 35 (40.7%) 1 (1.2%)
CLIP score n (%) 1 2 3 4	42 (47%) 36 (42%) 7 (8.4%) 1 (1.2%)
HAP score n (%) 1 2 3 4	3 (3.5%) 24 (27.9%) 29 (33.7%) 30 (34.9%)
Okuda score n (%) 1 2 3	3 (3.5%) 79 (90.7%) 4 (4.7%)
MELD score*	6.0 (6.0-17.5)
AFP (ng/ml)* < 400 ng/ml ≥ 400 ng/ml	4147 (0-60500) 49 (57%) 37 (43%)
Bilirubin total (µmol/l)*	12.4 (3.9–43.5)
Albumin (g/l)*	39.7 (22.9–47.0)

*ALBI* albumin-bilirubin, *AFP* alpha-fetoprotein, *AIH* autoimmune hepatitis, *ASH* alcoholic steatohepatitis, *BCLC* Barcelona Clinic Liver Cancer, *CLIP* Cancer of the Liver Italian Program, *CTP* Child- Turcotte-Pugh-, *HAP* hepatoma arterial-embolization prognostic, *HBV* hepatitis B virus, *HCV* hepatitis C virus, *MASLD* metabolic dysfunction-associated steatotic liver disease, *MELD* Model of End Stage Liver Disease, *PBC* primary biliary cholangitis

*median (range)

Additional treatments during follow-up (49/86) included systemic therapy (*n* = 15), TACE (*n* = 11), TARE and systemic (*n* = 3), TARE (*n* = 2), TACE and systemic therapy (*n* = 6), TARE and TACE (*n* = 3), TARE and liver transplantation (LTX) (*n* = 2), external radiation (*n* = 3), resection (*n* = 1) or combinations of three or more different treatment approaches (*n* = 2) and one patient unknown.

### Factors Associated with Survival After TARE

**Fig. 1:**
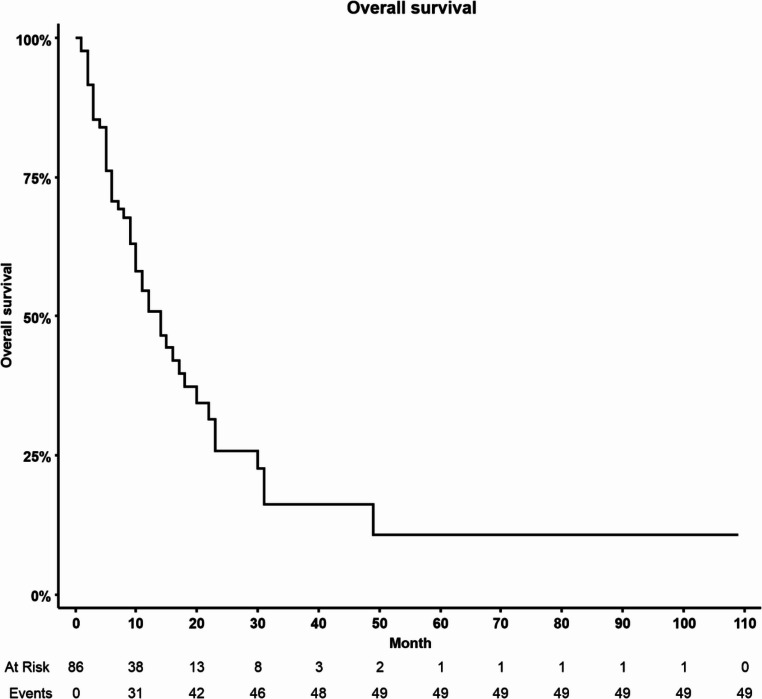
Kaplan-Meier curve showing overall survival for all patients with hepatocellular carcinoma after transarterial radioembolization

The median overall survival (OS) after TARE was 9 (range, 0-109) months (Fig. 1). The estimated overall survival rate was 10.7% ± 6.0%.

Comparing ALBI, CTP, CLIP, Okuda, BCLC and MELD scores, only the ALBI score (hazard ratio (HR) = 3.83 [95% confidence interval (CI): 1.85–7.291] *p* = 0.0003), the CLIP score (HR = 1.70 [95% CI: 1.09–2.64] *p* = 0.019) and the Okuda score showed statistically significant association with OS (HR = 7.71 [95% CI: 2.16–27.52] *p* = 0.002) (Table 2).

**Table 2:** Multivariable analysis of the association of baseline factors with overall survival after transarterial radioembolization (TARE)

Parameter	Univariate analysis			Multivariable analysis		
	HR	95% CI	*p*-value	HR	95% CI	*p*-value
ALBI	2.41	1.28–4.55	**0.007**	3.83	1.85–7.91	**0.0003**
HAP	1.100	0.78–1.54	0.596			
CLIP	1.81	1.27–2.57	0.001	1.70	1.09–2.64	0.019
CTP A B	REF 2.41	0.72-8.00	0.153			
BCLC	1.01	0.58–1.75	0.972			
Okuda	9.21	2.73–31.12	**0.0004**	7.71	2.16–27.52	**0.002**
MELD	1.06	0.97–1.17	0.181			
Age	1.01	0.98–1.03	0.674			
Sex Female Male	REF 0.95	0.43–2.13	0.907			
MVI No Yes	REF 1.32	0.74–2.37	0.348			
Multiple nodules No Yes	REF 0.98	0.52–1.85	0.938			
AFP [ng/ml]	1.00	1.00–1.00	**0.003**	1.00	1.00–1.00	0.792

*AFP* alpha-fetoprotein, *ALBI* albumin-bilirubin, *BCLC* Barcelona Clinic Liver Cancer, *CI* confidence interval, *CTP* Child- Turcotte-Pugh-, *CLIP* Cancer of the Liver Italian Program, *HAP* hepatoma arterial-embolization prognostic, *HR* hazard ratio, *MELD* Model of End Stage Liver Disease, *MVI* macrovascular infiltration, *REF* reference. Ordinal variables: CLIP, HAP, Okuda, BLCL, continuous variables: ALBI, Age, MELD, AFP

### Time-Dependent ROC Analyses

**Fig. 2:**
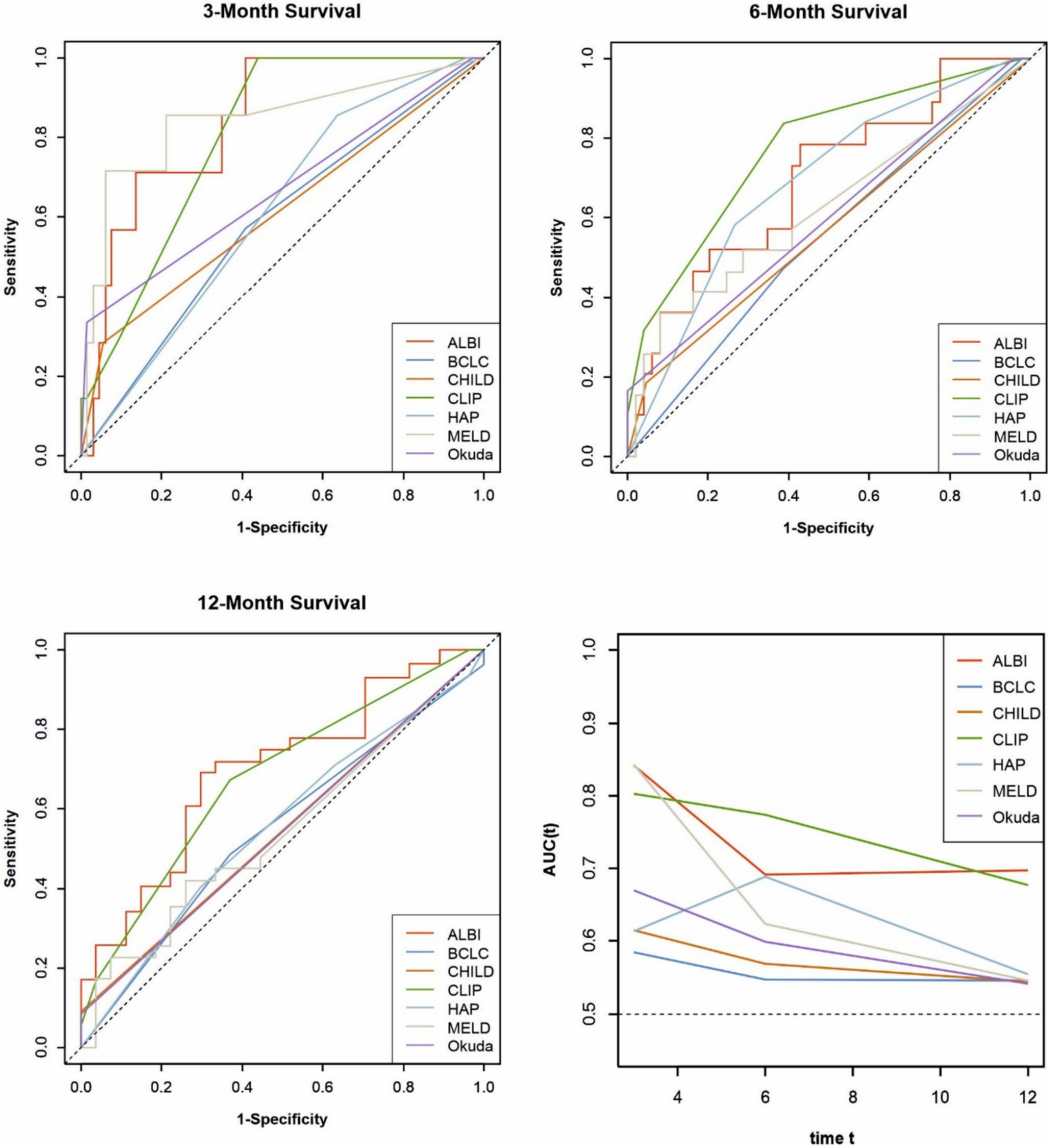
Time-dependent AUC of different scores for 3-, 6- and 12-month survival after transarterial radioembolization using time-dependent receiver operating characteristic (ROC) analyses. ALBI: albumin-bilirubin, AUC: area under the receiver operating curve, BCLC: Barcelona Clinic Liver Cancer, CLIP: Cancer of the Liver Italian Program, HAP: hepatoma arterial-embolization prognostic, MELD: Model of End Stage Liver Disease

In time-dependent ROC analyses, the ALBI and CLIP scores are most appropriate to predict the 12-, 6- and 3-month survival after TARE with area under the receiver operating curve (AUROCs) of 69.7–84.1% and 67.7–80.4%, respectively (Fig. 2 and Table S2). The AUROCs of both scores were not significantly different. However, the predictive probability of all scores decreases over time.

### Prediction of 3-Month Survival

In univariate Cox regression analysis, the majority of all tested prognostic scores (ALBI (*p* = 0.003), CLIP (*p* = 0.002), HAP (*p* = 0.053), Okuda (*p* = 0.006), MELD (*p* = 0.0006) and values AFP (*p* = 0.004), showed significant association with 3-months survival after TARE. After multivariable testing of parameters with p-values below 0.1 in univariate analysis, only the ALBI score remained independently associated with 3-month survival with an HR of 5.56 (95% CI: 1.17–26.35, *p* = 0.031) (Table S3).

### Prediction of 6-Month Survival

In univariate analysis, the prognostic scores ALBI (*p* = 0.031), CLIP (*p* = 0.0004), Okuda (*p* = 0.016) and AFP (*p* = 0.005) were significantly associated with 6-months survival. The ALBI score (HR = 4.12 [95%CI: 1.60-10.62] *p* = 0.003) and Okuda score (HR = 6.23 [95% CI: 1.67–23.27] *p* = 0.007) showed a significant association with 6-months survival in multivariable testing (Table S4).

### Prediction of 12-Month Survival

In multivariable Cox regression analysis of prognostic factors associated with 12-month survival, the ALBI score and Okuda remained statistically significant (HR = 4.28 [95% CI: 1.94–9.43] *p* = 0.0003 and HR = 8.16 [95% CI: 2.36–28.25] *p* = 0.0009, respectively) (Table S5).

**Fig. 3:**
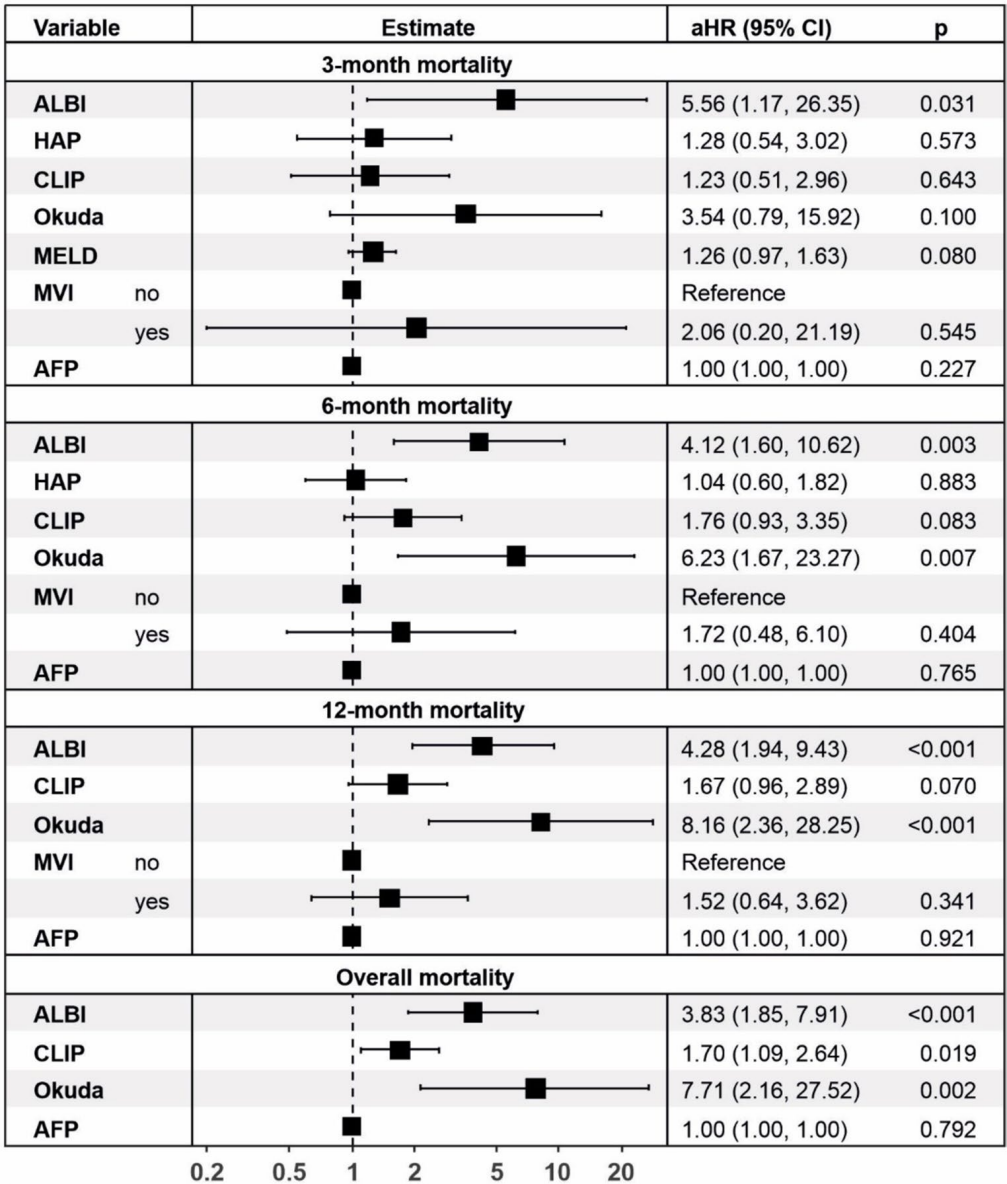
Cox regression analyses for 3-month, 6-month, 12-month and overall mortality in patients after transarterial radioembolization (TARE). aHR: adjusted hazard ratio, ALBI: albumin-bilirubin, AFP: alpha-fetoprotein, CI: confidence interval, CLIP: Cancer of the Liver Italian Program, HAP: hepatoma arterial-embolization prognostic, MELD: Model of End Stage Liver Disease, MVI: macrovascular infiltration

A summary of the multivariable Cox regression analysis results of all time points is shown in Fig. 3.

### Changes in Serum Parameters and ALBI Before and After TARE

In the total population, there were significant changes in gamma-glutamyl transpeptitase (GGT) levels (*p* = 9.31 × 10^− 10^), bilirubin levels (*p* = 5.43 × 10^− 6^), alkaline phosphatase (AP) levels (*p* = 0.017), platelet count (*p* = 0.003) and Quick (*p* = 0.040) between baseline and week 4 after TARE (Table S6).

The median ALBI score was also significantly higher after TARE (*p* = 0.0002) and during follow-up observation reflecting worsend liver function (Fig. S2).

## Discussion

Our study is the first to directly compare multiple established prognostic scores in predicting outcomes for patients with HCC undergoing TARE. For this, we have encompassed those scoring systems focused solely on liver function (ALBI, MELD, CTP) and those incorporating tumor characteristics (Okuda, CLIP, HAP, BCLC). The central finding of our analysis was that the ALBI score was the only score with a statistically significant association in all time frames (3-, 6-, and 12-month survival and overall survival). In the multivariable Cox regression analysis of 12-month survival, both the ALBI and Okuda scores showed both statistical significances.

The ALBI score remained the only factor in the multivariable cox regression independently associated with 3-month survival. The AUROC analysis for 3-, 6-, and 12-month survival demonstrated an appropriate predictive potential of OS for both the ALBI and CLIP scores.

When focusing on short-term survival after TARE, we made some noteworthy observations. The ALBI score significantly worsened in the 12 weeks following TARE (*p* = 4.5 × 10⁻⁵) (Fig. S1). In addition to albumin and the ALBI score, which demonstrated significant changes after TARE, platelet count and prothrombin time (quick) also showed significant alterations, suggesting a deterioration in liver function following treatment (Table S6).This indicates that monitoring liver function prior to TARE, particularly in patients with low ALBI scores, is crucial for determining whether liver-directed therapy is appropriate.

The findings of our study suggest that liver function, as measured by the ALBI score, is a key predictor of overall survival, influencing future treatment decisions. Tumor burden seems subordinate in locoregional therapy like TARE where a positive evaluation of TARE ensures a definitive tumor treatment.

A Spanish single-center real-life study demonstrated that the ALBI score was the only factor significantly associated with overall survival (HR 5.03) under systemic therapy with atezolizumab and bevacizumab (atezo/bev) [26]. Similarly, in a study conducted by Unome et al., ALBI deterioration within three months of starting atezo/bev was significantly linked to decreased overall survival (*p* = 0.005) [27]. Regarding TARE, a retrospective monocentric study found ALBI to be superior to the CPT classification in predicting overall survival, although no other prognostic scoring systems were included in that analysis [20].

In another study, Gui et al. showed that ALBI score provided better survival discrimination than the CPT class in HCC patients treated with TARE, supporting our own findings [28]. Conversely, Gelardi et al. observed that in treatment-naïve patients, ALBI score did not predict outcomes, while the presence and severity of portal vein thrombosis (PVT) had a significant prognostic impact [29]. Nevertheless, ALBI still outperformed the CPT classification. One limitation of this study is the unusually long median overall survival of 54 months, given that patients were primarily from BCLC stages B and C.

Cabibbo et al. showed that in the IMbrave150 study, early decompensation of liver function was associated with a 7.5-fold increased risk of death, a higher risk than that associated with radiological progression (HR 5.9) [30]. This post-hoc analysis underscores that early liver decompensation (within 3 months of treatment initiation and in the absence of tumor progression) has a profound independent prognostic effect on survival. Liver decompensation and tumor progression must therefore be considered competing risks, particularly in the first three months of follow-up [31]. Our findings are in accordance with a recent multi-center real-world database study, which showed superiority of ALBI score versus CTP score, suggesting a shift toward more nuanced liver function assessment in real world clinical practice [32]. The high proportion of patients with CPT A may habe limited the discriminatory performance of the Child-Pugh based scores. Evaluation of liver functional reserve e.g. through ALBI Score is crucial in patient selection not only for locoregional but systemic therapy and future trial design in HCC treatment.

### Tumor Burden and Combined Scores

Scores that incorporate both liver function and tumor burden, such as the CLIP and Okuda scores, did not show superior predictive value compared to the ALBI score. The CLIP score, which includes CTP stage (liver function), tumor morphology, AFP levels, and portal vein thrombosis (PVT), demonstrated strong predictive accuracy in the ROC curve analysis for 3-, 6-, and 12-month survival, similar to ALBI. However, the inclusion of these extra tumor parameters did not translate into an improved survival prediction in multivariable analysis compared to ALBI alone. This strongly suggests that the predictive power of the CLIP score in this cohort was largely driven by its liver function component.

This study has several limitations. First, it is a single-center study, which may limit the generalizability of our findings. Second, the retrospective design is inherently subject to bias. Additionally, a substantial number of patients had to be excluded due to missing imaging data, which may have affected the representativeness of the study population and may reduce the significance of our findings. Given the 10-year study period, temporal changes in diagnostic work-up, treatment algorithms, and supportive care may have introduced heterogeneity and selection bias that could have affected the study results.

In conclusion, the superior predictive performance of the ALBI score over models including tumor characteristics (like tumor burden or PVT) highlights the importance of hepatic functional reserve as the key determinant of outcomes following locoregional liver cancer treatments like TARE. The clinical relevance of the ALBI score for patient selection for TARE should be validated in prospective analyses.

## Supplementary Information

Below is the link to the electronic supplementary material.

Supplementary Material 1 (PDF 150 KB)

Supplementary Material 2 (DOCX 158 KB)

## Data Availability

The data are available from the corresponding author on reasonable request.

## Abbreviations

AFP: alpha-fetoprotein
AIH: autoimmune hepatitis
ALBI: albumin-bilirubin
ALT: alanine aminotransferase
AP: alkaline phosphatase
ASH: alcoholic steatohepatitis
AS: aspartate aminotransferase
Atezo/Bev: Atezolizumab/Bevacizumab
AUROC: area under the receiver operating curve
BCLC: Barcelona Clinic Liver Cancer
CI: confidence interval
CLIP: Cancer of the Liver Italian Program
CPT: Child-Pugh-Turcotte
CT: computed tomography
GBq: gigabecquerel
GGT: gamma-glutamyl transpeptitase
HAP: hepatoma arterial-embolization prognostic
HBV: hepatitis B virus
HCC: hepatocellular carcinoma
HCV: hepatitis C virus
HR: hazard ratio
MASLD: metabolic dysfunction-associated steatotic liver disease
MELD: Model of End Stage Liver Disease
mRECIST: modified Response Evaluation Criteria in Solid Tumors
MRI: magnetic resonance imaging
MVI: macrovascular infiltration
OS: overall survival
PBC: primary biliary cholangitis
PET: positron emission tomography
PVT: portal vein thrombosis
RFS: recurrence-free survival
ROC: receiver operating characteristic
Suppl: supplementary
TACE: transarterial chemoembolization
TARE: transarterial radioembolization
Tbl: table
Y-90: yttrium-90

## Declarations

### Approval of the research

The study was conducted according to the guidelines of the Declaration of Helsinki and was approved by the Ethics Committee of Medical Research of the University of Leipzig (protocol code 132/23-ek, 09.05.2023) for studies involving humans.

### Registry and Registration No. of the study/trail

N/A.

### Animal studies

N/A.

### Research involving recombinant DNA

N/A.
