# Supplementary material for: Prognostic Value of Liver Function and Tumor Burden Scores in Patients with HCC After Transarterial Radioembolization – Retrospective Single Center Analysis
Source: J Gastrointest Cancer. 2026 Aug 13;57(1):184. doi: 10.1007/s12029-026-01572-1 (PMC13473070; doi:10.1007/s12029-026-01572-1)
Supplement: Supplementary file 1 [file 12029_2026_1572_MOESM1_ESM.pdf]

STROBE Statement—checklist of items that should be included in reports of observational studies

	Item No.	Recommendation	Page No.	Relevant text from manuscript
**Title and abstract**	1	(*a*) Indicate the study's design with a commonly used term in the title or the abstract	1	We changed the title into "Prognostic value of liver function and tumor burden scores in patients with HCC after transarterial radioembolization - retrospective single center analysis"
		(*b*) Provide in the abstract an informative and balanced summary of what was done and what was found	3	see section abstract
**Introduction**				
Background/rationale	2	Explain the scientific background and rationale for the investigation being reported	5-6	See section introduction
Objectives	3	State specific objectives, including any prespecified hypotheses	6	See section introduction, last paragraph
**Methods**				
Study design	4	Present key elements of study design early in the paper	6-7	see section materials and methods, title adapted
Setting	5	Describe the setting, locations, and relevant dates, including periods of recruitment, exposure, follow-up, and data collection	6-7	see section materials and methods
Participants	6	(*a*) *Cohort study*—Give the eligibility criteria, and the sources and methods of selection of participants. Describe methods of follow-up<br>*Case-control study*—Give the eligibility criteria, and the sources and methods of case ascertainment and control selection. Give the rationale for the choice of cases and controls<br>*Cross-sectional study*—Give the eligibility criteria, and the sources and methods of selection of participants	6-7	Retrospective single center data. Eligibility described under section materials and methods. Follow up restrained to retrospective character of the study.
		(*b*) *Cohort study*—For matched studies, give matching criteria and number of exposed and unexposed	n.a.	

		Case-control study—For matched studies, give matching criteria and the number of controls per case		
Variables	7	Clearly define all outcomes, exposures, predictors, potential confounders, and effect modifiers. Give diagnostic criteria, if applicable	page 6-7 + 13-14	Described and provided in section materials and methods and discussion
Data sources/ measurement	8*	For each variable of interest, give sources of data and details of methods of assessment (measurement). Describe comparability of assessment methods if there is more than one group	6-7	Patient data were retrospectively extracted from the institutional electronic medical records.
Bias	9	Describe any efforts to address potential sources of bias		Retrospective mono-centric study with inherent bias.
Study size	10	Explain how the study size was arrived at		Please see flow chart Fig S1 page 25 of the manuscript. Retrospectively analysed data set.

Continued on next page

Quantitative variables	11	Explain how quantitative variables were handled in the analyses. If applicable, describe which groupings were chosen and why	page 6-7	Quantitative variables are presented as median and range, before and after TARE, not normally distributed
Statistical methods	12	(*a*) Describe all statistical methods, including those used to control for confounding	page 6-7	Statistical analyses described under section materials and methods
		(*b*) Describe any methods used to examine subgroups and interactions	page 6-7	Kruskal-Wallis test, post-hoc Dunns test, Friedmann test, Wilcoxon test, Cox regression analysis, variance inflation factor
		(*c*) Explain how missing data were addressed	page 6-7	
		(*d*) *Cohort study*—If applicable, explain how loss to follow-up was addressed<br>*Case-control study*—If applicable, explain how matching of cases and controls was addressed<br>*Cross-sectional study*—If applicable, describe analytical methods taking account of sampling strategy		Loss to follow up wasn't addressed due to the retrospective character of the study. If no data post-TARE were provided, patients were excluded.
		(*e*) Describe any sensitivity analyses		Not performed
**Results**				
Participants	13*	(a) Report numbers of individuals at each stage of study—eg numbers potentially eligible, examined for eligibility, confirmed eligible, included in the study, completing follow-up, and analysed	page 8-11, 25	See flow chart Fig S1 and under section results.
		(b) Give reasons for non-participation at each stage		Patients were excluded due to missing data or no follow up
		(c) Consider use of a flow diagram	page 25 Fig S1	
Descriptive data	14*	(a) Give characteristics of study participants (eg demographic, clinical, social) and information on exposures and potential confounders	page 16 Table 1	All patient characteristics are summarized in Table 1
		(b) Indicate number of participants with missing data for each variable of interest		All patient characteristics are summarized in Table 1 – percentage of patients for each variable are provided.

		(c) *Cohort study*—Summarise follow-up time (eg, average and total amount)	page 8	retrospectively assessed follow up data
Outcome data	15*	*Cohort study*—Report numbers of outcome events or summary measures over time	*page 8-12*	section results
		Case-control study—Report numbers in each exposure category, or summary measures of exposure	*n.e.*	
		Cross-sectional study—Report numbers of outcome events or summary measures	*n.e.*	
Main results	16	(*a*) Give unadjusted estimates and, if applicable, confounder-adjusted estimates and their precision (eg, 95% confidence interval). Make clear which confounders were adjusted for and why they were included	Page 8, 11-13, 18, Table S2-S4	Values and estimates of confidence intervals are provided (95% CI)
		(*b*) Report category boundaries when continuous variables were categorized		see table 1 and 2
		(*c*) If relevant, consider translating estimates of relative risk into absolute risk for a meaningful time period	n.a.	

Continued on next page

Other analyses	17	Report other analyses done—eg analyses of subgroups and interactions, and sensitivity analyses	n.a.	
**Discussion**				
Key results	18	Summarise key results with reference to study objectives	see discussion page 14	"In conclusion, the superior predictive performance of the ALBI score over models including tumor characteristics (like tumor burden or PVT) highlights the importance of hepatic functional reserve as the key determinant of outcomes following locoregional liver cancer treatments like TARE."
Limitations	19	Discuss limitations of the study, taking into account sources of potential bias or imprecision. Discuss both direction and magnitude of any potential bias	see discussion page 15	"First, it is a single-center study, which may limit the generalizability of our findings. Second, the retrospective design is inherently subject to bias. Additionally, a substantial number of patients had to be excluded due to missing imaging data, which may have affected the representativeness of the study population and may reduce the significance of our findings."
Interpretation	20	Give a cautious overall interpretation of results considering objectives, limitations, multiplicity of analyses, results from similar studies, and other relevant evidence	page 14 and 15 section discussion	Requested interpretation and discussion provided.
Generalisability	21	Discuss the generalisability (external validity) of the study results	n.a.	No external cohort was assessed.
**Other information**				
Funding	22	Give the source of funding and the role of the funders for the present study and, if applicable, for the original study on which the present article is based	n.a.	This study was funded by the Department of Hepatology at the University Hospital Leipzig.
