# Supplementary material for: Prognostic Value of Liver Function and Tumor Burden Scores in Patients with HCC After Transarterial Radioembolization – Retrospective Single Center Analysis
Source: J Gastrointest Cancer. 2026 Aug 13;57(1):184. doi: 10.1007/s12029-026-01572-1 (PMC13473070; doi:10.1007/s12029-026-01572-1)
Supplement: Supplementary file 2 [file 12029_2026_1572_MOESM2_ESM.docx]

**Table S1**. **Prognostic scores for hepatocellular carcinoma: Okuda, CLIP, ALBI, HAP**

| **Okuda** | Each one point for >50% tumor; ascites yes; albumin <30g/dl; bilirubin ≥3mg/dl | | | | |
| --- | --- | --- | --- | --- | --- |
| Stage | I | II | III |  |  |
| points | 0 | 1-2 | 3-4 |  |  |
|  | | | |  |  |
| **CLIP** |  | | | |  |
| Stage | I | II | III | IV |  |
| Total points | 0-1 | 2-4 | 5-6 | 7-8 |  |
|  |  |  |  |  | |
| points | 0 | 1 | 2 |  |  |
| *Child* | A | B | C |  |  |
| *AFP* | <400ng/ml | ≥400ng/ml |  |  |  |
| *PVT* | No | yes |  |  |  |
| *Tumor* | Solitary nodule ≤50% | Multiple nodules ≤50% | >50% |  |  |
|  |  |  |  |  |  |
| **ALBI** | Calculated: ALBI Score = (log_10_ Bilirubin in μmol/L × 0.66) + (Albumin in g/L × (-0.085)) | | | | |
| ALBI grade 1 (lowest mortality risk) | | | ALBI score ≤2.60 | |  |
| ALBI grade 2 (intermediate mortality risk) | | | ALBI score -2.60 < and ≤1.39 | |  |
| ALBI grade 3 (highest mortality risk) | | | ALBI score >-1.39 | |  |
|  |  |  |  |  |  |
| **HAP** | Each one point for albumin >36g/dl; bilirubin >17µmol/l; AFP>400ng/ml; tumor >7cm | | | | |
| stage | A1 | B2 | C3 | D4 |  |
| points | 0 | 1 | 2 | >2 |  |

**Table S2. Time-dependent calculation of the area under the receiver operating curve (AUROCs) for 3-, 6- and 12-months**

| **Score** | **Time** | **AUROC (%)** | **95% CI** |
| --- | --- | --- | --- |
| **ALBI** | 3 | 84.13 | 71.86-96.41 |
|  | 6 | 69.17 | 55.19-83.16 |
|  | 12 | 69.71 | 56.31-83.11 |
| **BCLC** | 3 | 58.47 | 39.33-77.60 |
|  | 6 | 54.76 | 41.64-67.88 |
|  | 12 | 54.57 | 41.66-67.47 |
| **CHILD** | 3 | 61.51 | 44.42-78.61 |
|  | 6 | 56.90 | 46.80-66.99 |
|  | 12 | 54.49 | 49.58-59.39 |
| **CLIP** | 3 | 80.35 | 70.18-90.52 |
|  | 6 | 77.40 | 65.82-88.85 |
|  | 12 | 67.67 | 55.36-79.97 |
| **HAP** | 3 | 61.32 | 43.17-84.14 |
|  | 6 | 68.84 | 52.52-85.16 |
|  | 12 | 55.50 | 37.91-73.09 |
| **MELD** | 3 | 84.28 | 66.42-102.14 |
|  | 6 | 62.38 | 45.56-77.20 |
|  | 12 | 54.56 | 41.16-67.97 |
| **Okuda** | 3 | 67.05 | 48.47-88.65 |
|  | 6 | 59.91 | 50.00-69.81 |
|  | 12 | 54.18 | 48.86-59.51 |

ALBI: albumin-bilirubin, BCLC: Barcelona Clinic Liver Cancer, CI: confidence interval, CLIP: Cancer of the Liver Italian Program, HAP: hepatoma arterial-embolization prognostic, HR: hazard ratio, MELD: Model of End Stage Liver Disease,

**Table S3: Uni- and multivariable analyses of factors associated with 3-month survival after transarterial radioembolization (TARE)**

| Parameter | Univariate analysis | | | Multivariable analysis | | |
| --- | --- | --- | --- | --- | --- | --- |
|  | **HR** | **95% CI** | **p-value** | **HR** | **95% CI** | **p-value** |
| ALBI | 4.68 | 1.68-13.04 | **0.003** | 5.56 | 1.17-26.35 | **0.031** |
| HAP | 2.19 | 0.99-4.85 | 0.053 | 1.28 | 0.54-3.02 | 0.573 |
| CLIP | 2.24 | 1.36-3.68 | **0.002** | 1.23 | 0.51-2.96 | 0.643 |
| CTP  A  B | 3.60 | 0.78-16.69 | 0.102 |  |  |  |
| BCLC | 0.83 | 0.28-2.45 | 0.738 |  |  |  |
| Okuda | 8.38 | 1.83-38.45 | **0.006** | 3.54 | 0.79-15.92 | 0.100 |
| MELD | 1.28 | 1.11-1.47 | **0.0006** | 1.26 | 0.97-1.63 | 0.080 |
| Age | 0.99 | 0.94-1.05 | 0.761 |  |  |  |
| Sex  Female  Male | REF  0.51 | 0.14-1.88 | 0.311 |  |  |  |
| MVI  No  Yes | REF  6.70 | 0.86-51.89 | 0.069 | REF  1.72 | 0.48-6.10 | 0.545 |
| Multiple nodules  No  Yes | REF  0.99 | 0.27-3.66 | 0.989 |  |  |  |
| AFP [ng/ml] | 1.00 | 1.00-1.00 | **0.004** | 1.00 | 1.00-1.00 | 0.227 |
| AFP: alpha-fetoprotein, ALBI: albumin-bilirubin, BCLC: Barcelona Clinic Liver Cancer, CI: confidence interval, CLIP: Cancer of the Liver Italian Program, CTP: Child- Turcotte-Pugh-, HAP: hepatoma arterial-embolization prognostic, HR: hazard ratio, MELD: Model of End Stage Liver Disease, MVI: macrovascular infiltration, REF: reference. Ordinal variables: CLIP, HAP, Okuda, BLCL, continuous variables: ALBI, Age, MELD, AFP | | | | | | |

**Table S4: Uni- and multivariable analyses of factors associated with survival less than 6 month after transarterial radioembolization (TARE)**

| Parameter | Univariate analysis | | | Multivariable analysis | | |  |
| --- | --- | --- | --- | --- | --- | --- | --- |
|  | **HR** | **95% CI** | **p-value** | **HR** | **95% CI** | **p-value** |  |
| ALBI | 2.43 | 1.08-5.43 | **0.031** | 4.12 | 1.60-10.62 | 0.003 |  |
| HAP | 1.60 | 0.96-2.68 | 0.074 | 1.04 | 0.60-1.82 | 0.883 |  |
| CLIP | 1.95 | 1.35-2.84 | **0.0004** | 1.76 | 0.93-3.35 | 0.083 |  |
| CTP  A  B | REF  2.52 | 0.74-8.62 | 0.141 |  |  |  |  |
| BCLC | 1.42 | 0.65-3.09 | 0.376 |  |  |  |  |
| Okuda | 4.21 | 1.31-13.49 | 0.016 | 6.23 | 1.367-23.27 | 0.007 |  |
| MELD | 1.11 | 0.97-1.27 | 0.121 |  |  |  |  |
| Age | 1.00 | 0.96-1.04 | 0.910 |  |  |  |  |
| Sex  Female  Male | REF  0.86 | 0.29-2.53 | 0.784 |  |  |  |  |
| MVI  No  Yes | REF  2.84 | 0.97-8.37 | 0.058 | REF  1.72 | 0.48-6.10 | 0.404 |  |
| Multiple nodules  No  Yes | REF  0.65 | 0.28-1.54 | 0.333 |  |  |  |  |
| AFP [ng/ml] | 1.00 | 1.00-1.00 | **0.005** | 1.00 | 1.00-1.00 | 0.765 |  |
| AFP: alpha-fetoprotein, ALBI: albumin-bilirubin, BCLC: Barcelona Clinic Liver Cancer, CI: confidence interval, CLIP: Cancer of the Liver Italian Program, CTP: Child- Turcotte-Pugh, HAP: hepatoma arterial-embolization prognostic, HR: hazard ratio, MELD: Model of End Stage Liver Disease, MVI: macrovascular infiltration, REF: reference. Ordinal variables: CLIP, HAP, Okuda, BLCL, continuous variables: ALBI, Age, MELD, AFP | | | | | | | |

**Table S5: Uni- and multivariable analyses of factors associated with 12-month survival after transarterial radioembolization (TARE)**

| Parameter | Univariate analysis | | | Multivariae analysis | | |  |
| --- | --- | --- | --- | --- | --- | --- | --- |
|  | **HR** | **95% CI** | **p-value** | **HR** | **95% CI** | **p-value** |  |
| ALBI | 2.63 | 1.35-5.14 | **0.004** | 4.28 | 1.94-9.43 | **0.0003** |  |
| HAP | 1.30 | 0.88-1.92 | 0.186 |  |  |  |  |
| CLIP | 2.11 | 1.4-3.09 | **0.0003** | 1.67 | 0.96-2.89 | 0.070 |  |
| CTP  A  B | REF  2.41 | 0.72-8.00 | 0.153 |  |  |  |  |
| BCLC | 1.02 | 0.54-1.92 | 0.959 |  |  |  |  |
| Okuda | 9.21 | 2.73-31.12 | **0.0004** | 8.16 | 2.36-228.25 | **0.0009** |  |
| MELD | 1.10 | 0.98-1.23 | 0.115 |  |  |  |  |
| Age | 1.00 | 0.97-1.03 | 0.921 |  |  |  |  |
| Sex  Female  Male | REF  0.76 | 0.31-1.82 | 0.533 |  |  |  |  |
| MVI  No  Yes | REF  2.08 | 0.97-4.45 | 0.059 | REF  1.52 | 0.64-3.62 | 0.341 |  |
| Multiple nodules  No  Yes | REF  0.72 | 0.35-1.47 | 0.363 |  |  |  |  |
| AFP [ng/ml] | 1.000 | 1.00-1.00 | **0.002** | 1.00 | 1.00-1.00 | 0.921 |  |
| AFP: alpha-fetoprotein, ALBI: albumin-bilirubin, BCLC: Barcelona Clinic Liver Cancer, CI: confidence interval, CLIP: Cancer of the Liver Italian Program, CTP: Child- Turcotte-Pug-, HAP: hepatoma arterial-embolization prognostic, HR: hazard ratio, MELD: Model of End Stage Liver Disease, MVI: macrovascular infiltration, REF: reference. Ordinal variables: CLIP, HAP, Okuda, BLCL, continuous variables: ALBI, Age, MELD, AFP | | | | | | | |

**Table S6:** **Blood parameters before and at week 4 after transarterial radioembolization (TARE).** Values are shown as median and range. Comparison was performed with paired Wicoxon test.

| Parameter | Before TARE | | Week 4 after TARE | |  |
| --- | --- | --- | --- | --- | --- |
|  | **Median** | **Range** | **Median** | **Range** | **p-value** |
| ALT [µkat/L] | 0.60 | 0.17-4.05 | 0.60 | 0.19-2.65 | 0.462 |
| AST [µkat/L] | 0.87 | 0.30-5.09 | 0.91 | 0.32-5.21 | 0.968 |
| AP [µkat/L] | 2.35 | 1.07-15.86 | 2.25 | 0.97-15.58 | **0.017** |
| GGT [µkat/L] | 3.27 | 0.62-25.08 | 2.43 | 0.37-22.69 | **9.31x10^-10^** |
| Leucocytes [10^9^/L] | 6 | 2.3-20.2 | 5.9 | 1.30-1.6 | 0.357 |
| Hemoglobin [gm/dL] | 8.2 | 3.6-10.7 | 8.1 | 3.3-10.7 | 0.245 |
| Platelets [10^9^/L] | 141 | 40-391 | 149 | 46-397 | **0.003** |
| Albumin [g/L] | 39.7 | 22.9-47.0 | 39.6 | 25.4-50.0 | 0.448 |
| Bilirubin [µmol/L] | 12.4 | 3.9-43.5 | 15 | 4.5-67.0 | **5.43x10^-6^** |
| Creatinine [µmol/L] | 80 | 44-211 | 79.5 | 41-362 | 0.304 |
| Quick | 83 | 50-126 | 81 | 39-118 | **0.040** |
| AFP [ng/mL] | 4147 | 0-60500 | 99 | 4-60500 | 0.396 |
| ALBI score | -2.74 | -3.47--1.29 | -2.63 | -3.39—1.22 | **1.91x10^-4^** |
| AFP: alpha-fetoprotein, ALBI: albumin-bilirubin, ALT: alanine aminotransferase, AP: alkaline phosphatase, AST: aspartate aminotransferase, GGT: gamma-glutamyl transpeptitase, TARE: transarterial radioembolization | | | | | |

**Fig. S1** Patient with transarterial radioembolization (TARE) included in the study.

**Fig. S2** Course of ALBI score after transarterial radioembolization (TARE). Friedman test (p=1.37x10^-4^) and posthoc paired Wilcoxon test were performed.
